# Encapsulating *Calendula arvensis* (Vaill.) L. Florets: UHPLC-HRMS Insights into Bioactive Compounds Preservation and Oral Bioaccessibility

**DOI:** 10.3390/molecules28010199

**Published:** 2022-12-26

**Authors:** Marika Fiorentino, Simona Piccolella, Claudia Gravina, Adriano Stinca, Assunta Esposito, Michelina Catauro, Severina Pacifico

**Affiliations:** 1 Department of Environmental, Biological and Pharmaceutical Sciences and Technologies, University of Campania “Luigi Vanvitelli”, Via Vivaldi, 43, 81100 Caserta, Italy; 2 Department of Engineering, University of Campania “Luigi Vanvitelli”, Via Roma, 29, I-813031 Aversa, Italy

**Keywords:** *Calendula arvensis*, field marigold, polyphenols, triterpene saponins, simulated in vitro digestion, UHPLC-HRMS

## Abstract

Wild edible plants, once consumed in times of famine or for health purposes, today represent an interesting dietary supplement, aimed at enriching local dishes and/or formulating healthy nutraceutical products. In fact, the broad content of different, and diversely bioactive, specialized metabolites therein suggests new scenarios of use which, in order to be as functional as possible, must maximize the bioactivity of these compounds while preserving their chemistry. In this context, based on a recent investigation on the metabolic profile of the organs of *Calendula arvensis* that highlighted that florets are abundant in flavonol glycosides and triterpene saponins, the freeze-drying encapsulation of their alcoholic extract (FE) into maltodextrin (MD) was investigated. FE-MD chemical composition was evaluated using Fourier Transform InfraRed spectroscopy (FTIR), while ultra-high performance liquid chromatography coupled with high-resolution tandem mass spectrometry (UHPLC-HRMS/MS) techniques were employed to unravel FE compound preservation also during in vitro simulated digestion. The establishment of H-bonds between FE compounds and MD hydroxyl groups was in line with FE-MD biocompatibility in Caco-2 cells, while in vitro digestion mostly affected structural integrity and/or diversity. Flavonol compounds underwent deglycosylation and demethylation, while deacylation, beyond oxidation, involved triterpene saponins, which massively preserve their aglycone core.

## 1. Introduction

The culture of consuming alimurgic plants dates back to ancient times. Nowadays, although their usage has been frequently undervalued, they are experiencing a renaissance [1,2] as a new trend in nutrition in contemporary European cuisine [3,4] and ecosystem service [5]. Alimurgic plants, also known as wild edible plants (WEPs), are recognized as a source of micro- and macro-nutrients for dietary requirements, as well as of bioactive compounds for a functional nutrition [6]. The scientific recognition of their healthy properties has been accompanied by different strategies aimed at increasing the conservation of biodiversity and promoting the rural areas where they grow [7,8]. The latter is in line with the goal of contributing to people’s local identity [9], which is largely made up of traditional cuisine, including cooking methods and the events during which they are consumed [2].

Among the WEPs, *Calendula arvensis* (Vaill.) L. (also known as field marigold) is a medicinal species used in the health field, and with an important place in popular tradition due to its pleasant sensory properties. In several Mediterranean regions, boiled leaves, basal rosettes, and tender shoots are consumed, including the aerial parts sometimes mixed with meatballs [10,11]. The flowers or inflorescences of *C. arvensis* are eaten raw in salads or used to make jams and even candied. In southern Italy the dried petals of *C. arvensis* are reported to flavor wine, which after being left in the sun for ten days becomes excellent vinegar [12]. *C. arvensis* is also used in a traditional ointment made from crushed flowers mixed with olive oil to treat wounds and mouth ulcers [13,14]. Recently, *C. arvensis* has also been reported for the Campania Region (Southern Italy), where in particular its florets and leaves, employed in salads or soups of local cuisine, have become part of the historical-cultural heritage of Cilento, Vallo di Diano, and Alburni National Park (PNVCDA), one of the most extensive protected areas in Italy [15]. The chemical composition of *C. arvensis’* six organs has been very recently investigated, providing a deep insight into their diversity in specialized metabolites, mainly polyphenols and triterpene saponins, which will hopefully open new scenarios for its rational nutraceutical use [16]. Indeed, these compounds are prone to lose their structural goodness based on environmental factors (e.g., light, heat, oxygen degradation) and processing conditions [17,18] Moreover, further instability characterizes their digestion process at the gastrointestinal level after ingestion, resulting in a decrease in the putative bioactivity, as the native amount in the plant matrix barely corresponds to the bioaccessible and bioavailable fraction [19,20]. 

In this context, strategies capable of safeguarding the specialized bioactive metabolites from harmful chemical alterations, also responsible for their reduced efficacy, must be pursued. Encapsulation, defined as a process involving the incorporation of a bioactive compound (called core) into an inert coating (called wall or carrier material), is one of the most convincing procedures aimed at protecting bioactive compounds or promoting their controlled release in those districts where they exert a beneficial effect [21,22]. Several encapsulation methods have been proposed, with freeze-drying tools representing a valuable method for encapsulating polar and thermolabile pure compounds and/or extracts [23,24]. In addition, various coatings have been applied; regardless of the wall material (e.g., polysaccharides, proteins, or lipids), certain characteristics must be met, such as being food-grade and biodegradable. Among the saccharides, maltodextrin (MD), a non-sweet nutrient mixture consisting of d-glucose-based oligomers and polymers, is an advantageous choice due to the high availability of raw material, high water solubility, and low viscosity [25,26].

In light of the above, herein the MD encapsulation process via freeze-drying was exploited as a valuable strategy to preserve the integrity and antioxidant activity of *C. arvensis* polar specialized compounds and to mitigate the degradation process in the gastro-intestinal tract during digestion. For this purpose, florets of field marigold harvested in PNVCDA preliminarily underwent ultrasound-assisted maceration in MeOH. Encapsulates were analyzed using Fourier Transform Infra-Red (FTIR) spectroscopy before and after an in vitro digestion test to investigate the nature of interactions established, as well as the effect of the digestion process on the maintenance of the structure capable of guaranteeing bioactivity. The deep chemical profiling was achieved by means of ultra-high performance liquid chromatography hyphenated to high-resolution tandem mass spectrometry (UHPLC-HRMS/MS) tools. Compound bioaccessibility at the end of the simulated digestion process was also assessed using UHPLC-HRMS/MS to obtain information about the occurrence of bioactive compounds that are potentially available for carrying out their activity. 

## 2. Results and Discussion

The encapsulation procedure of *C. arvensis* floret extract (FE) was carried out using 10% (*w*:*v*) maltodextrin (MD) as the wall material. The literature data on the different MD:core ratios have shown that an increase in this ratio is directly related to a higher encapsulation efficiency due to a higher viscosity, which hinders the migration of the compounds towards the surface [27]. Based on these previous findings, an MD:core ratio equal to 1:2 (*w*:*w*) was chosen. The FE-MD mixture was freeze-dried, producing a powder with good rehydration properties [28]. 

### 2.1. Encapsulation Efficiency and FTIR Analysis

The FE encapsulation efficiency in maltodextrin wall material was estimated by means of colorimetric assays, aimed at determining the amount of entrapped phenols and saponins inside the polysaccharide matrix compared to the amount located on the wall surface. In fact, a previous work [16], focused on the in-depth compositional analysis of *C. arvensis* organ methanolic extracts, revealed that florets are mainly composed of glycosylated flavonols, besides triterpene saponins, with a different abundance detected for each metabolite. Although the plant organ investigated herein was collected in a different geographic location but with a similar land use habitat, it was reasonable to assume a similar chemical composition from a qualitative point of view. 

**Figure 1:**
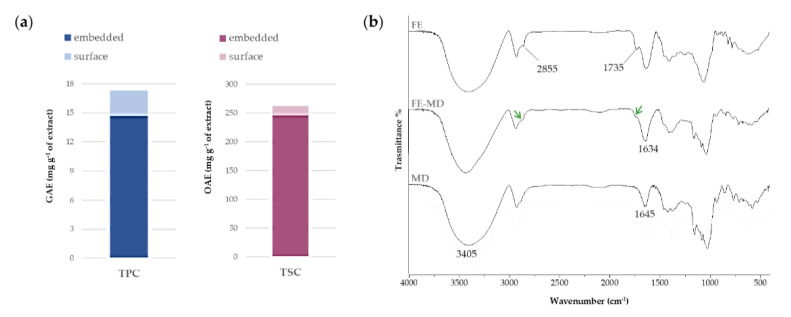
(**a**) Total Phenol Content (TPC), expressed as mg GAE per g of extract, and Total Saponin Content (TSC), expressed as mg OAE per g of extract. The total content of each class of compounds takes into account relative surface and embedded contents. Values are reported as the mean ± SD of three independent measurements. (**b**) FTIR spectra of floret extract (FE), floret extract encapsulated in maltodextrin (FE-MD), and pure maltodextrin (MD). Green arrows focus on CH_2_ asymmetric and symmetric stretching, and estereal C=O symmetric stretching in FE-MD (please refer to the text).

The results of the Folin–Ciocalteau assay for determining total phenol content (TPC), expressed as mg of gallic acid equivalents (GAEs) per g of extract, underlined a seven-fold lower content of these metabolites on the surface with respect to the internal core (2.6 ± 1.0 mg GAE), leading to a phenol encapsulation efficiency equal to about 85%. Saponins embedded in the matrix accounted for 245.48 ± 14.7 mg of oleanolic acid equivalents (OAEs) per g of extract. This value was about 15-fold higher when compared to the surface amount (Figure 1a). The overall encapsulation efficiency (%) was estimated to be 93.2 ± 6.0. These results suggest a strong interaction between the bioactive compounds and the polysaccharide matrix, leading us to hypothesize their protection during in vitro simulated digestion.

The FTIR spectroscopy allowed us to unravel the nature of the bonds established in encapsulates between the MD wall material and the floret extract. In Figure 1b FTIR spectra recorded before and after the encapsulation procedure are reported. All spectra showed a broad band in the range 3600–3100 cm^−1^, attributable to the symmetric and asymmetric stretching of free hydroxyl groups, as well as of those employed in intra- and inter-molecular H-bonds, of carbohydrates, phenols, and polyphenols [29]. In particular, the OH vibrations shifted to lower (11–12 cm^−1^) wavelength values compared with the MD signal, indicating the formation of new H-bonds between extract constituents and the polysaccharidic carrier. 

**Figure 2:**
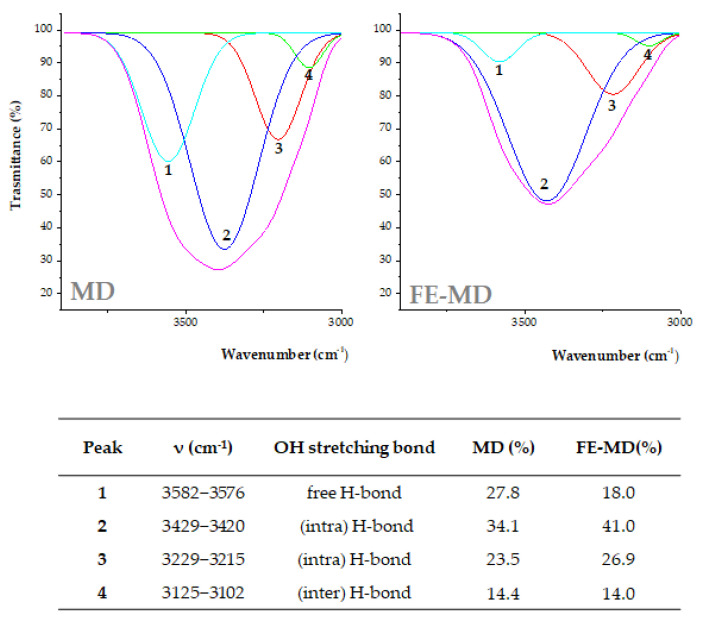
OH stretching band deconvolution FTIR spectra from MD and FE-MD using the Gaussian model. Percentage area peaks from deconvoluted signals are also reported.

The deconvolution spectra (Figure 2) allowed us to better investigate the contribution of H-bonds with respect to free OH groups. Four components were revealed in the Gaussian deconvolution analysis. The peak at higher cm^−1^ values (3582–3578 cm^−1^) was assigned to OH stretching vibration of free OH groups in carbohydrates, phenols, and alcohols. 

The peaks at 3429–3420 and 3229–3215 cm^−1^ were consistent with intra-molecular H-bonds, whereas the band at 3125–3102 cm^−1^ was likely due to the inter-molecular hydrogen bonds formed between the wall carrier and encapsulated extract. The percentage area of each OH stretching component was calculated (Figure 2). The free H-bond area was higher in MD and decreased when new H-bonds were established in FE-MD. This result also suggests the increase in H-bonds in the floret-based encapsulate [30]. 

Moreover, the bands at 2929 and 2845 cm^−1^ in Figure 1 were in accordance with CH_2_ asymmetric and symmetric stretching, whereas the peak at 1735 cm^−1^ was attributable to the stretching vibration of ester C=O groups. In the FE-MD spectrum the decrease and broadening of these peaks (labeled by green arrows) highlighted the encapsulation achievement in the MD wall with interaction that likely involved these functional groups. The bare maltodextrin spectrum shows its characteristic peaks in the fingerprint region. Indeed, the peaks at 1154 cm^−1^ and 1077 cm^−1^, and 1024 cm^−1^, were related to C-O stretching and the angular deformation of =CH and =CH_2_ bonds, respectively, typical of carbohydrate units, whereas the band at 1645 cm^−1^ could be assigned to deformation vibrations of –OH groups [31,32]. 

### 2.2. Evaluation of Cytotoxicity on the Caco-2 Cell Line

An MTT test was performed on the Caco-2 cell line after 3 h exposure time to investigate a potential cytotoxic effect exerted by the formulations in a time span that is slightly higher than that required by a typical simulated intestinal digestion phase.

**Figure 3:**
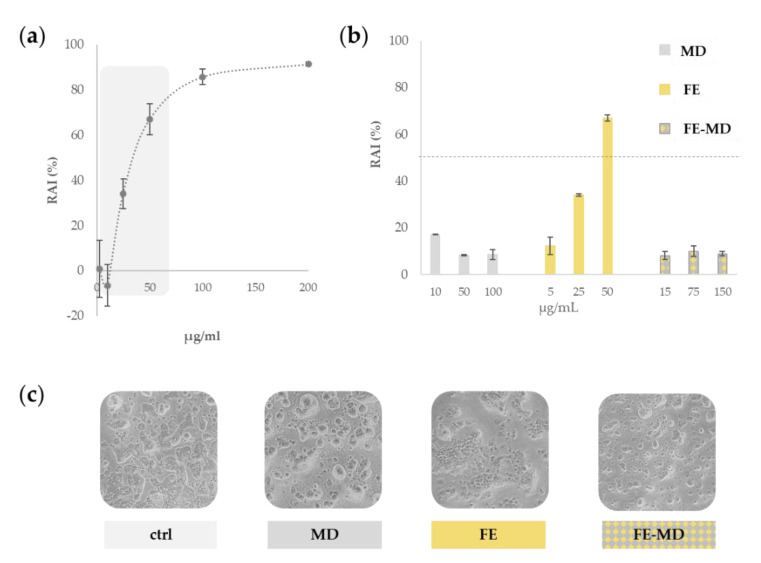
(**a**) Preliminary assessment of redox activity inhibition (RAI %) of floret extract (FE) towards Caco-2 cells. (**b**) Comparison of RAI % due to FE, FE-MD formulation, and MD. (**c**) Representative images of untreated Caco-2 cell line, and cells treated with maltodextrin (MD), floret extract (FE), and FE-MD at the highest dose. Images were acquired with an Inverted Phase Contrast Brightfield Zeiss Primo Vert Microscope.

A dose–response curve was prepared for FE in a wide concentration range, from 2.5 to 200 μg/mL (Figure 3a). 

The results clearly indicated that floret extract had a dose-dependent cytotoxic effect, inhibiting significantly the cell mitochondrial redox activity (RAI) by more than 50%, when the concentration of 50 μg/mL was tested. Thus, this concentration was selected as the highest dose level of the core of MD encapsulates, and the role of the polysaccharide wall in the cell protection was investigated. Indeed, as depicted in Figure 3b, the field-marigold-MD formulation did not display cytotoxicity reduction, and its RAI value never exceeded 25%. This finding is in accordance with a high biocompatibility, as also revealed by morphologically observing treated cells (Figure 3c). 

Maltodextrin, as an inert polysaccharide, is generally considered as safe by the US Food and Drug Administration [33]. In fact, it was previously reported that cell growth is usually not affected by the presence of this polymer, which acts only as a carrier [34], while exerting a protective role towards oxidation and increasing the stability of the encapsulated compounds [35]. A similar positive trend was found when microencapsulating stevia ethanolic extract in maltodextrin (ratio 1:2, *w*:*w*), which showed viability higher than 80%, strongly reducing the extract’s cytotoxicity even at the highest tested dose [36].

### 2.3. UHPLC-QqTOF-MS/MS Analysis before In Vitro Digestion

The chemical composition of *C. arvensis* alcoholic floret extract herein investigated, based on UHPLC-QqTOF-MS/MS data, is summarized in Table 1 and briefly discussed below.

Apart from compound **1**, likely corresponding to a [M + Cl^−^]^−^ adduct of a dihexose, the detected specialized metabolites were phenols, polyphenols, and triterpene saponins, as expected from the results of the TPC and TSC tests and previously published chemical profiles [16].

#### 2.3.1. Phenols and Polyphenols

Compounds **2**–**4** and **13** were quinic acid esters, whose hydroxycinnamoyl moiety consisted of caffeoyl in **2** and **3** (at *m*/*z* 353.0880, C_16_H_18_O_9_) and feruloyl in **4** (at *m*/*z* 367.1043, C_17_H_20_O_9_). In their HR-MS/MS spectra, base peaks at *m*/*z* 191.056 derived from the neutral loss of dehydrated hydroxycinnamoyl moieties were observed, whereas the fragment ion at *m*/*z* 193.0505 in **4** was in agreement with ferulate ion. In particular, based on the fragmentation pattern and comparison with pure reference compounds, compounds **2** and **3** were identified as the geometrical isomers of 5-O-caffeoyl quinic acid [37]. Furthermore, the TOF-MS/MS spectrum of compound **13**, showing a deprotonated molecular ion at *m*/*z* 515.1196, was in accordance with 3,5-di-O-caffeoylquinic acid [37]. 

Compounds **5**–**12** and **14**–**20** were tentatively identified as flavonol glycosides, with quercetin, kaempferol, and isorhamnetin as aglycone units. In particular, metabolites **5**–**9** and **12** were glycosylated quercetin derivatives whose elution order in reversed-phase chromatography was in accordance with a gradual decrease in polarity, mainly ascribable to the glyconic moiety. Thus, compounds **5**–**7** and **9** were quercetin diglycosides, bearing a dihexose, a hexosylpentose, and hexosyldeoxyhexoses, lost as dehydrated units (−324, −294, and −308 Da, respectively) [16]. The intensity ratio between the flavonol radical deprotonated ion (at *m*/*z* 300.027) and the related deprotonated aglycone (at *m*/*z* 301.035) allowed us to hypothesize the same glycosylation position (at C-3 hydroxyl group). The deprotonated molecular ion detected for metabolite **8** (*m*/*z* 463.0882, calc. mass) and the formula calculated therefrom (C_21_H_20_O_12_) suggested the occurrence of a quercetin hexoside, whereas compound **12** was putatively its malonyl derivative. In fact, the detection of fragment ions at *m*/*z* 505.0988 and 463.0885 in the TOF-MS/MS experiment was consistent with sequential losses of CO_2_ (44 Da) and C_2_H_2_O units (42 Da) from the precursor molecular ion to give quercetin hexoside (Figure S1). The latter compound was previously identified in *C. officinalis* flowers and petals and in *C. morifolium* [38], but, to the best of our knowledge, never in *C. arvensis*.

Based on our previous MS data, compounds **11** and **14** were identified as two isomers of kaempferol-3-O-hexoside, whereas compound **15** was in line with kaempferol-7-O-rutinoside [16].

Metabolites **10** and **16**–**20** were isorhamnetin glycosides. In particular, compounds **16** and **18**, sharing the same molecular formula (C_28_H_32_O_16_), were tentatively identified as isorhamnetin-3-O-hexosyldeoxyhexoside and its 7-O isomer, respectively. The intensity ratio between the [aglycone–H]^−^ (at *m*/*z* 315.051) and the [aglycone–H]^•−^ (at *m*/*z* 314.043) ions, formed following the neutral loss of the disaccharide moiety (−308/309 Da), suggested a different glycosylation position. Compound **10** (at calc. *m*/*z* 639.1567, C_28_H_32_O_16_) was likely isorhamnetin-3-O-dihexoside, while metabolites **17** and **19** were two isomers of monohexosyl methoxyflavonol. Finally, the deprotonated compound **20** at *m*/*z* 563.1042, whose neutral losses resembled those observed for compound **12**, was tentatively identified as the malonyl derivative of isorhamnetin-3-O-hexoside (Figure S1), and not reported before in field marigold florets.

#### 2.3.2. Triterpene Saponins

Recently, deeply investigating the fragmentation pathways of *C. arvensis* triterpene saponins, we provided some useful information for their HR-MS and MS/MS straightforward chemical characterization in complex mixtures [16]. Herein, we applied those guidelines to identify all the detected compounds belonging to this class (**21**–**38**), among which seven saponins were not identified in our previous work. Thus, their identification is discussed herein in detail, providing hypothesized fragmentation pathways as supplementary material. 

Saponins **21**–**26**, **28**, and **36** were echinocistic acid glycosides. Compound **21** with formula C_47_H_74_O_19_ and [M − H]^−^ at *m*/*z* 941.4790 (4.1 ppm mass error) was tentatively identified as 3-O-(pentosyl)hexuronidyl 28-O-echinocystic acid hexosyl ester. Its fragmentation pattern resembles that described for calendasaponin B, differing in the presence of a pentose residue instead of a hexose, linked to the hexuronidyl moiety. Briefly, the [M − H]^−^ ion, following the neutral loss of 162.05 Da, generated the fragment ion at *m*/*z* 779.4207. The latter provided an ion at *m*/*z* 717.4229 through the concerted loss of (44 + 18 = 62) Da on the hexuronidyl unit, or of the pentosyl unit (−150 Da) to provide the fragment ion at *m*/*z* 629.3699. Alternatively, from the ion at *m*/*z* 717.4229, following the neutral loss of 132 Da (pentose-H_2_O), the fragment at *m*/*z* 585.3797 was obtained. The deprotonated echinocystic acid at *m*/*z* 471.3480 could be finally revealed (Figure S2). 

Compound **23** (C_42_H_66_O_15_, at *m*/*z* 809.4369) differed from the previous one in the absence of the pentosyl residue. Thus, it was tentatively identified as 3-O-hexuronidyl 28-O-echinocystic acid hexosyl ester, as reported by Fiorentino et al. [16]. Metabolite **25** seemed to be a derivative of the previous one bearing an acetylhexosyl instead of a hexosyl residue at the C-28 position. In fact, the first neutral loss of 42 Da from the deprotonated molecular ion gave rise to the ion at *m*/*z* 809.4329, and then the fragmentation pattern proceeded in a similar way (Figure S3). Neutral losses of 144 Da from compounds **26** (at *m*/*z* 953.4792, C_48_H_74_O_45_) and **36** (at *m*/*z* 791.4235, C_42_H_64_O_14_) were in accordance with a hydroxymethylglutaryl moiety on the molecular skeleton and differed in a hexose unit, as previously described [16].

Unlike the above-mentioned saponins, compounds **22**, **24**, and **28** were detected in the TOF-MS spectrum as formate adducts ([M + HCOO]^−^), giving evidence of the absence of a primarily acid site (neither free triterpene carboxylic group nor hexuronic acid). Thus, metabolite **24** was tentatively identified as 3-O-hexosyl 28-O-echinocystic acid hexosyl ester. In fact, the fragment ions at *m*/*z* 633.4030 and 587.3957 were generated following the neutral loss of the dehydrated hexosyl unit (−162 Da) at C-28 and the sequential loss of 46 Da. Then, the loss of the second hexosyl moiety (as 180 Da) gave rise to the ion at *m*/*z* 407.3348 (Figure S4). Moreover, following the same rationale, saponins **22** and **28** were tentatively identified as two isomers of 3-O-dihexosyl 28-O-echinocystic acid hexosyl ester (e.g., calendulastellaside B), whose fragmentation pathway is reported in Figure S5.

Saponins **27**, **29**–**35**, **37**, and **38** were recognized as oleanolic acid glycosides, differing in the number and the nature of saccharidic units. Also within this subclass, for some compounds formate adducts were detected. In particular, TOF-MS and TOF-MS/MS spectra of compounds **27**, **30**, **33**, and **38** were in accordance with the presence of 3-O-trihexosyl, dihexosyl (e.g., arvensoside A), and two monohexosyl derivatives of 28-O-oleanolic acid hexosyl ester, respectively, previously reported in *C. arvensis* organs [16].

All the other oleanolic acid glycosides showed deprotonated molecular ions. TOF-MS/MS fragmentation of saponins **29** and **31** (calc. mass at *m*/*z* 925.4802, C_47_H_74_O_18_) suggested the occurrence of two isomers of in which a pentose, a hexose, and a hexuronic acid were linked to oleanolic acid. Following the fragmentation route described for saponin **21**, they were putatively assigned to 3-O-(pentosyl)hexuronidyl 28-O-oleanolic acid hexosyl esters. In both cases, the loss of the sugar units generated the deprotonated oleanolic acid at *m*/*z* 455.3531 (calc. mass) (Figure S6). Grati et al. [39] very recently reported oleanolic saponins in *C. aegyptiaca* fruits, providing a tentative structure in which both the pentose and the hexose were linked at the C-2 and C-3 positions of the uronic acid. Compound **32** was likely 3-O-hexuronidyl 28-O-oleanolic acid hexosyl ester (e.g., calenduloside F) or calenduloside G [39], whereas in metabolite **34** an acetylhexosyl moiety was supposed to be esterified at the C-28 position of 3-O-hexuronidyl oleanolic acid, in accordance with Fiorentino et al. [16]. The [M − H]^−^ ion detected for compound **37** (at *m*/*z* 763.4303, C_41_H_64_O_13_) fragmented, losing a pentose unit (−150 Da) and generating the ion at *m*/*z* 613.3767, which in turn provided the oleanolate ion by the neutral loss of 158 Da, as described above (Figure S7). Thus, it was putatively identified as 3-O-(pentosyl)hexuronidyl oleanolic acid.

Finally, the fragmentation pattern of saponin **35** was consistent with the hydroxymethylglutaryl derivative of **34**. In fact, after the loss of 144 Da from the deprotonated molecular ion (at *m*/*z* 937.4802, calc. mass) the two TOF-MS/MS spectra were almost superimposable.

### 2.4. Bioaccessibility Assessment of Encapsulated Bioactive Compounds by In Vitro Digestion

FE-MD underwent in vitro simulated digestion to obtain information about how the encapsulation in maltodextrin was able to influence the bioaccessibility in the gastro-intestinal tract of floret bioactive compounds. 

**Figure 4:**
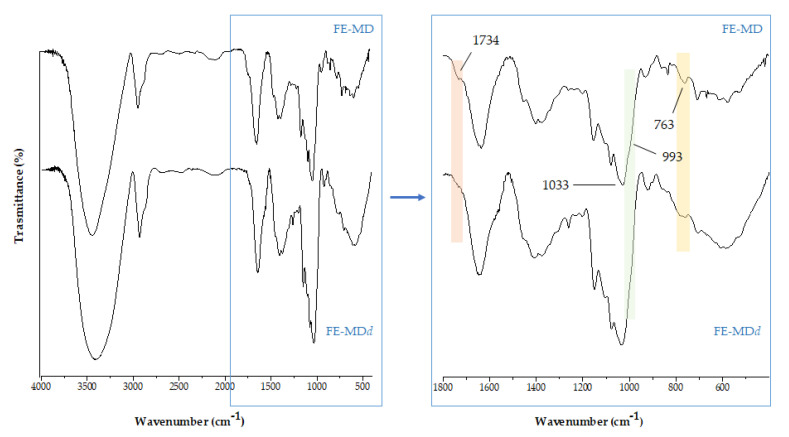
The comparison of FTIR spectra of floret encapsulate before (FE-MD) and after in vitro digestion (FE-MDd).

FTIR spectra acquired after in vitro digestion gave preliminary insights about its capability to induce structural changes in encapsulates. In Figure 4 the zoomed region in the range 1800–500 cm^−1^ is reported for the digested formulation (FE-MDd), also in comparison with the native (undigested) sample. The most detectable change regards a decrease in the bands at 1734(5) cm^−1^ and 763 cm^−1^, attributable to the stretching vibration of carbonyl groups and the bending vibration of C-H in hydroxycinnamic acids and other phenolic acids [40], which could be in accordance with their lower occurrence in digestates. Moreover, the shoulder band at 993 cm^−1^, characteristic of C-O-C stretching vibration in polysaccharides, weakened, leading us to hypothesize the partial cleavage of α(1→4) glycosidic bonds within the maltodextrin wall [41]. 

UHPLC-HRMS tools allowed us to obtain more specific information about the fate of individual compounds at the end of the digestion process. However, a massive matrix effect was revealed in total ion current chromatograms of digesta, due to the presence of degradation products of maltodextrin, which eluted at the lowest retention time, resulting in metabolite ion suppression (data not shown). In order to avoid this problem, samples deriving from in vitro simulated digestion underwent a liquid/liquid extraction using n-butanol as the extracting solvent. The organic phases were collected and analyzed with hyphenated UHPLC and HRMS techniques compared to undigested samples. 

A preliminary insight evidenced that (poly)phenols were massively affected by digestion degradation, whereas triterpenes were more preserved. Indeed, Bottcher et al. [42] reported that when saponins are dissolved in an aqueous system, such as digestion buffers, they are able to form a viscoelastic network through intermolecular hydrogen bonds with neighboring sugar residues, which is able to protect the phytocomplex embedded in the maltodextrin coating. 

**Figure 5:**
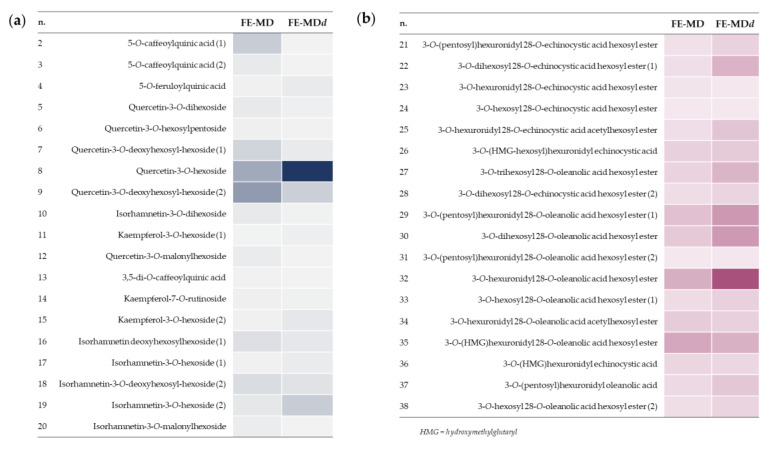
Heatmaps of polyphenols (**a**) and triterpene saponins (**b**) in floret encapsulates before and after in vitro digestion (FE-MD and FE-MDd, respectively).

FE-MDd was the object of an in-depth UHPLC-HRMS study to analyze the bioaccessibility and putative biotransformations of its bioactive compounds during in vitro digestion. The relative amount of polyphenols and triterpene saponins before and after in vitro simulated digestion could be easily visualized through the heatmap analysis, reported in Figure 5. 

**Figure 6:**
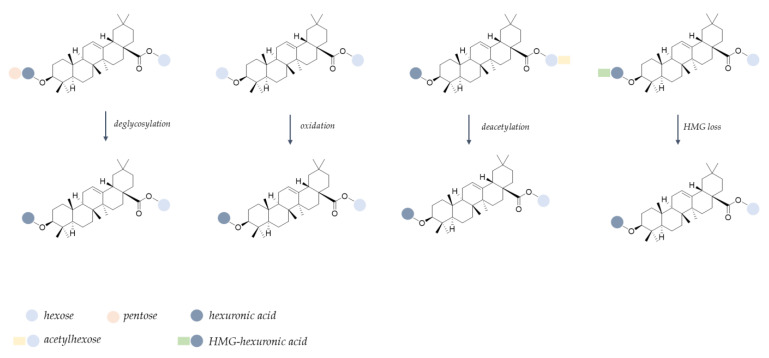
Proposed biotransformation pattern for triterpene saponins during in vitro simulated digestion (HMG = hydroxymethylglutaryl).

In particular, the hydroxycinnamoyl derivatives appeared particularly vulnerable, in accordance with hypotheses deriving from FTIR spectra. In fact, poor traces of 5-O-caffeoylquinic acid isomers, 5-O-feruloylquinic acid, as well as dicaffeoylquinic acid, were detected in the FE-MDd sample, in line with the ester bond cleavage that has been reported to occur in the simulated gastric acidic environment [43,44]. Flavonoid glycosides were partially degraded, also as a consequence of biotransformation reactions. In particular, a decrease in quercetin malonylhexosyl and diglycosyl derivatives, such as quercetin-3-O-dihexoside, quercetin-3-O-hexosylpentoside, and rutin, were observed in the digestate following deacylation and glycosidic bond hydrolysis to provide the quercetin 3-O-hexoside. Indeed, the latter was 2.5-fold more abundant in digested floret encapsulate compared with the undigested one. Similarly, signals assigned to diglycosyl derivates, as well as to the malonyl-hexosyl conjugate of isorhamnetin, strongly decreased, putatively giving rise to isorhamnetin-3-O-hexoside isomers (Figure S8). The occurrence of deacylation and deglycosylation reactions has been recently described as metabolic steps of flavonoid O-glycosides both at the gastric and at the intestinal level [45]. Moreover, it is worthy of note that quercetin 3-O-hexoside could also be generated following isorhamnetin 3-O-hexoside demethylation reactions at C-3′ position, or, alternatively, by hydroxylation of kaempferol 3-O-hexoside [46]. In line with the previous hypothesis, deacetylation and deglycosylation reactions, together with the loss of the hydroxymethylglutaryl moiety and hexose oxidation, were also assumed to explain structure modifications in triterpene saponins, leading to the formation of 3-O-hexuronidyl 28-O-oleanolic acid hexosyl ester (Figure 6).

### 2.5. Evaluation of Radical-Scavenging Capacity (RSC, %)

It is well known that the bioactivity of a compound closely depends on its chemical structure. The scenario is further complicated when a phytocomplex is considered and not pure molecules, as synergic or inhibiting effects could contribute to the obtained results. Thus, the bioactivity could be strongly affected by structural changes occurring during digestion steps.

**Figure 7:**
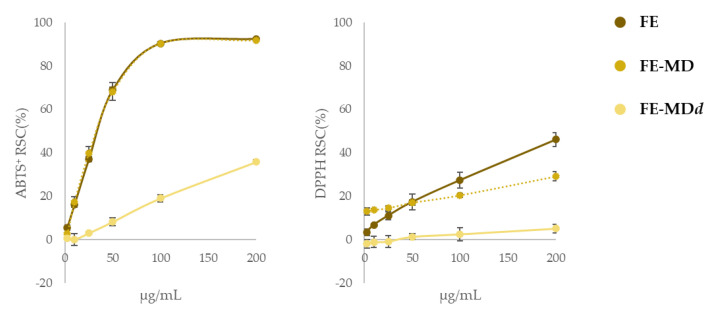
Radical-scavenging activity (RSC%) of floret extract (FE) in comparison with its encapsulate (FE-MD) and digested formula (FE-MDd) using DPPH and ABTS tests. Values reported are the mean ± SD of three independent measurements.

With this awareness, FE samples were tested to assess their radical-scavenging capacity against DPPH^•^ and ABTS^•+^ and the results were compared to FE-MD encapsulates, to evaluate the activity maintenance, and also to samples derived from the end of the simulated digestion procedure (FE-MDd) (Figure 7).

The radical-scavenging efficiency of encapsulates against ABTS^•+^ was superimposable to that recorded for the naked extract, proving that the encapsulation process did not affect the capacity of bioactive compounds entrapped in the maltodextrin matrix to neutralize the presence of the radical probe. Considering that both ABTS and DPPH tests have been explained based on a mix of SET (Single Electron Transfer) and HAT (Hydrogen Atom Transfer) mechanisms, with ABTS radicals more prone to the former, and DPPH to the latter, it was not surprising that responses to the DPPH test were much lower than the previous ones. In fact, the formation of H-bonds between the core and the MD wall in encapsulates could be responsible for the compounds’ reduced attitude as H donors. 

After the in vitro digestion a considerable reduction in the radical-scavenging activity was observed, again more pronounced vs. DPPH^•^. In this case, in line with data reported by Tarko et al. [47], the scenario is also worsened by the loss of a large portion of the polyphenols in the intestinal phase (only 10% of the total amount survived to the simulated digestion). 

## 3. Materials and Methods

### 3.1. Plant Collection and Extraction 

*Calendula arvensis* plants were collected in December 2021 at Ascea (Salerno, Italy), located in Cilento, Vallo di Diano, and Alburni National Park (PNCVDA) (40°09′45.2″ N 15°12′26.8″ E). A voucher specimen has been deposited in the Herbarium Austroitalicum (IT) of the Department of Environmental, Biological, and Pharmaceutical Sciences and Technologies of the University of Campania ‘Luigi Vanvitelli’ (Caserta, Italy). Fresh plants organs were dissected, immediately stored in liquid nitrogen, and transferred to the laboratory. Among them, after lyophilization, florets underwent a defatting extraction step in *n*-hexane and then were sonicated (Branson Ultrasonics^TM^ Bransonic^TM^ M3800-E; Danbury, CT, USA) in methanol to collect polar bioactive compounds. The alcoholic extract thus obtained was dried using a rotary evaporator.

### 3.2. Floret Extract Encapsulation

**Figure 8:**
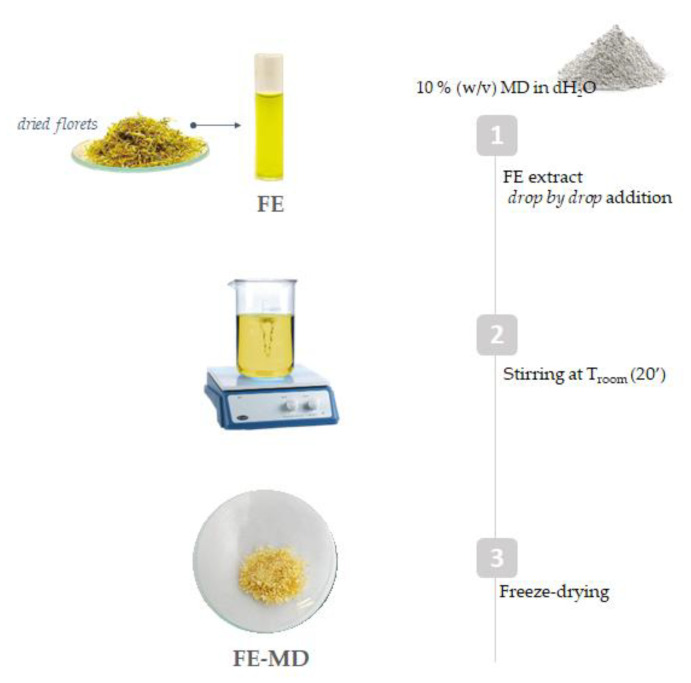
Encapsulation scheme applied to field marigold floret extract (MD = maltodextrin; FE = floret extract).

Maltodextrin (MD, DE 16.5–19.5) powder (1.2 g) was dissolved in 12 mL of distilled water. Floret extract (FE; 600 mg) was solubilized in distilled water and then added drop by drop to the maltodextrin solution, reaching a FE:MD ratio of 1:2, under continuous stirring at 25 °C. The mixture was stored at −80 °C for 12 h and then was cryo-dried for 48 h using the FTS-System Flex-dry^TM^ instrument 75 (SP Scientific, Stone Ridge, NY, USA). Finally, the obtained powdered sample was stored at 4 °C, sheltered from light, until use (Figure 8).

#### 3.2.1. Surface and Total Phenol Content in Encapsulates

Total phenol content (TPC) was determined according to the Folin–Ciocalteau method, as well as the surface phenol content. Briefly, for TPC, 2.5 and 5.0 mg of FE-MD were mixed with 2.25 mL of sodium carbonate (7.5% *w*/*v*) and 0.25 mL of Folin–Ciocalteau reagent. The mixtures were shaken for 3 h in the dark at room temperature. The absorbance was read at 765 nm using a Synergy spectrophotometer (Biotek, Winooski, VT, USA). Three replicates of three aliquots (*n* = 3) of each sample (in total, 3 × 3 measurements) were analyzed. A calibration curve of gallic acid was built up (0.78–50 µg/mL) and data were expressed as mg of gallic acid equivalents (GAEs) per g of sample (mean ± SD). For surface phenol content, FE-MD powder was mixed with ethyl acetate (1.0 mL), vortexed for 60 s, and centrifuged at 3000× *g* for 10 min. The surface phenol content was measured following the same Folin–Ciocalteau method. The embedded phenol content was calculated by subtracting the surface phenol content from the TPC value [48].

#### 3.2.2. Surface and Total Saponin Content in Encapsulates

For the determination of total saponin content (TSC), 2.5 and 5.0 mg of FE-MD were mixed with 150 µL of isovanillin/glacial acetic acid (5% *w*/*v*) and 0.5 mL of perchloric acid. The mixture was incubated at 60 °C for 45 min and cooled down in an ice bath. The absorbance was spectrophotometrically read at 550 nm. The total saponins were quantified using a standard curve of calibration of oleanolic acid and expressed as mg of oleanolic acid equivalents (OAEs) per g of sample [49]. For surface saponin content, FE-MD and LE-MD encapsulates were mixed with butanol (1.0 mL), vortexed for 60 s, and centrifuged for 10 min at 3000× *g*. The surface saponin content was then measured following the method applicated for TSC, while embedded saponin content was calculated as the difference between TSC and the surface saponin content.

#### 3.2.3. Evaluation of Encapsulation Efficiency 

The overall encapsulation efficiency (EE %) in maltodextrin was calculated by measuring the amounts of phenols and saponins entrapped in the formulation over the total phenols and saponins revealed [48,50], following the equation:

EE % = [(EPC + ESC)/(TPC + TSC)] × 100

where EPC and ESC are encapsulated phenol and saponin content, respectively, and correspond to (TPC—surface phenol content) and (TSC—surface saponin content) amounts.

### 3.3. FTIR Analysis of the Floret Encapsulate

Fourier transform infrared (FTIR) spectra of FE-MD encapsulates and floret extract, used as a reference together with bare MD, were recorded using a Prestige 21 system (Shimadzu, Milan, Italy) with a deuterated triglycine sulfate detector with potassium bromide windows (DTGS KBr). The wavenumber range was 4000–400 cm^−1^ with a resolution of 2 cm^−1^ (60 scans). A 200 mg KBr pellet disk of each sample (1% in KBr) was prepared using a Specac manual hydraulic press (Orpington, UK) equipped with a cylindrical holder. FTIR spectra were processed with IR solution software (v.1.60, Shimadzu, Milan, Italy). The multiple peak fit tools of OriginPro 2015 software (OriginLab Corp., Northampton, MA, USA) was employed for the deconvolution analysis of the OH stretching band (3600–3100 cm^−1^): number of peaks = 4; fitting function: Gaussian; R-square ≥ 0.999; no parameters were fixed for analysis. Chi-sqr tolerance reached 1 × 10^−9^ for all deconvoluted spectra.

### 3.4. Cytotoxicity Evaluation

The cytotoxic effect on the gastrointestinal tract were evaluated on the human colorectal adenocarcinoma epithelial cell line Caco-2 (ATCC^®^ HTB:37™, American Type Culture, Manassas, VA, USA), cultured in Dulbecco’s Modified Eagle’s Medium (DMEM) supplemented with 50.0 U/mL penicillin, 100.0 µg/mL streptomycin, and 10% fetal bovine serum, at 37 °C in a 5% CO_2_ humidified atmosphere [51,52]. A total of 2.5 × 10^4^ cells was seeded in each well of a 96-multiwell plate. After 24 h, cells were treated with different doses of the FE (2.5, 10, 25, 50, 100, and 200 µg/mL, final concentrations) for 3 h. Then, the inhibition of mitochondrial redox activity (RAI %) was determined with the MTT cell test. Based on the dose–response curves obtained for each sample, three different concentrations of the encapsulated extract were selected (15, 75, 150 µg/mL, containing 5, 25, 50 µg/mL of FE). Maltodextrin was used as the negative control sample, tested at the three different doses used in formulas to keep constant the 2:1 (MD:extract) ratio (10, 50, and 100 µg/mL). Two independent experiments were carried out with six replicate measurements in each of the three samples of the extract (in total, 6 × 3 measurements). Data were expressed as mean ± standard deviation (SD).

### 3.5. Bioaccessibility Study

The bioaccessibility of polyphenols and triterpene saponins in FE-MD was studied following the in vitro digestion procedure proposed by Minekus et al. [53]. Simulated salivary (SSF), gastric (SGF), and intestinal fluids (SIF) were employed for oral, gastric, and intestinal phases, respectively, prepared following the recommendations therein. One gram of FE-MD was suspended in SSF buffer, distilled water, and CaCl_2_ (0.3 M concentration in the mixture). A final ratio of encapsulates to SSF of 1:1 (*w*/*v*) was reached. After adding the porcine α-amylase enzyme (final concentration of 75 U/mL), the oral mixture was incubated for 2 min at 37 °C. The oral bolus was mixed with SGF with a final ratio of 1:1 *v*/*v*. The porcine pepsin enzyme and CaCl_2_ were added to achieve 2000 U/mL and 0.3 M in the final mixture, respectively. HCl (6 M) was added to obtain pH 3.0. The gastric phase step ended in 2 h under continuous stirring in the dark at 37 °C. Finally, the gastric chyme was mixed with SIF solution to obtain a 1:1 ratio *v*/*v* after the addition of pancreatin enzyme solution (100 U/mL based on trypsin activity, final concentration), 0.3 M CaCl_2_, bile salts (10 mM in the final mixture), and distilled water. NaOH 1 M solution was required to neutralize the mixture. The latter was incubated for 2 h under continuous stirring in the dark at 37 °C; after that the enzyme activity was blocked by immersion in liquid nitrogen.

Digested FE-MD was dissolved in water:butanol (1:1, *v*/*v*) and subjected to discontinuous liquid–liquid extraction (three cycles), in order to recover bioactive compounds from the maltodextrin wall material. Then, the obtained organic fractions were profiled using UHPLC-HRMS with the same experimental parameters used for native extracts.

### 3.6. UHPLC-ESI-MS and MS/MS Analyses 

The chromatographic separation was performed with a Shimadzu NEXERA UHPLC system (Shimadzu, Tokyo, Japan). A Luna^®^ Omega C18 (1.6 µm particle size, 50 × 2.1 mm i.d.) was employed, injecting 2.0 µL of each sample. The following linear gradient was built up, with mixtures of H_2_O (A) and CH_3_CN (B), both with 0.1% formic acid, as mobile phase: 0–1 min, 5% B; 1–7 min, 5→17.5% B; 7–9 min, 17.5→25% B; 9–18 min, 25→55% B; 18–20 min, 55→95% B; 20–21 min, 95% B. Then, the starting conditions were restored and maintained for 2 min for re-equilibration purposes. The flow rate was 500 µL/min. The MS analysis was carried out using a hybrid Q*q*TOF MS instrument, the AB SCIEX Triple TOF^®^ 4600 (AB Sciex, Concord, ON, Canada), equipped with a DuoSpray^TM^ ion source, which operated in the negative ElectroSpray (ESI) mode. The HR-MS method consisted of a full-scan TOF survey (accumulation time 250 ms, 100–1500 Da) and eight IDA (information-dependent acquisition) scans (accumulation time 100 ms, 80–1300 Da). The MS parameters were as follows: curtain gas 35 psi, nebulizer gas 60 psi, heated gas 60 psi, ion spray voltage −4.5 kV, interface heater temperature 500 °C. The Analyst^®^ TF 1.7 software was used for controlling the instrument, while data processing used PeakView^®^ software version 2.2. The TOF-MS/MS parameters used for phenols and polyphenols (e.g., flavonoids, hydroxycinnamoyl compounds) were: −100 V declustering potential (DP), −40 V collision energy (CE), and −15 V collision energy spread (CES). DP of −120 V, CE of −100 V, and CES of −25 V were set for obtaining insight into triterpene saponins.

### 3.7. Radical-Scavenging Capacity Assessment

The radical-scavenging capacity of FE, and of its encapsulate and the digestate, was evaluated against the DPPH (2,2-diphenyl-1-picrylhydrazyl) radical and ABTS [2,2′-azinobis-(3-ethylbenzothiazolin-6-sulfonic acid)] radical cation, as previously reported [54]. Briefly, after aqueous solubilization, the samples were added to the radical solution to reach the tested concentrations ranging from 2.5 to 200 μg/mL. Three replicate measurements for each sample (three for each concentration) were tested in reference to a blank, in which samples were replaced with distilled water. Trolox^®^ in the 2–32 μM final concentration range was employed as a positive standard. For FE-MD the tested doses referred to the floret concentration therein, taking into consideration the ratio FE (or LE):MD = 1:2. Data were expressed as mean ± standard deviation (SD) from three replicate measurements of three samples of the extract (in total, 3 × 3 measurements).

## 4. Conclusions

*Calendula arvensis* is locally used for health purposes, or for preparing traditional dishes. In order to promote the species’ valorization, allowing full usage of all its organs, the study of its polar bioactive compounds was previously investigated by means of extraction and fractionation strategies exploiting accelerated maceration with ultrasound.

The metabolic peculiarity of the floret alcoholic extract (FE) consisted of glycoside flavonols and triterpene saponins, mainly based on echinocystic and oleanolic acid, in line with its marked anti-radical capacity. In order to formulate an FE-based antioxidant preparation readily available for consumption, FE was screened for its cytotoxicity towards intestinal epithelial cells, and then encapsulated, at safe dose levels, into maltodextrin.

Encapsulation has proven to be an effective strategy to preserve the compounds in the extract, and the formation of complex-stabilizing hydrogen bonds was ascertained through FTIR spectroscopy, while the anti-radical efficacy was comparable to that exerted by FE. When in vitro simulated digestion was performed, a massive loss of the scavenging capacity was observed. This is largely due to the degradation of the flavonoid compounds, which are more responsive in DPPH and ABTS tube tests than triterpene saponins. The application of UHPLC HR MS/MS techniques downstream of the in vitro simulated digestion process shows that deacylation and oxidation reactions, with deglycosylation, impact the triterpenic saponins which, however, keep intact the aglycone core. This is not strictly true for flavonoids although deglycosylation and demethylation are key processes in the biotransformation of these compounds.

The constitution of a maltodextrin:FE formulation opens up to new investigations for its dedicated use in the nutraceutical, cosmeceutical, and phytoceutical sectors. The possibility of benefiting from non-invasive processing of the phytochemistry of a species realizes the optimization of short local supply chains that join together tradition and innovation.

## Figures and Tables

**Table 1:** Compounds tentatively identified in floret alcoholic extracts before in vitro digestion (base peaks in MS/MS experiments are reported in bold; RDB = Ring and Double Bonds, i.e., unsaturation degree; Rt = retention time). * Molecular ions detected as formate ([M + HCOO^−^]^−^) adducts. n.c. = not calculable.

Peak	Rt (min)	Molecular Formula	RDB	[M − H]^−^ Found (*m*/*z*)	[M − H]^−^ Calcd. (*m*/*z*)	Error (ppm)	MS/MS Fragments (*m*/*z*)	Tentative Assignment
**1**	0.288	C_12_H_22_O_11_	2.0	377.0890 [M + Cl^−^]^−^	n.c.	n.c.	377.0873, 341.1108, **179.0568**, 161.0461, 119.0354, 113.0252, 89.0248	Dihexose
**2**	2.677	C_16_H_18_O_9_	8.0	353.0880	353.0878	0.5	**191.0566**, 85.0295	5*-O-*caffeoylquinic acid (1)
**3**	3.126	C_16_H_18_O_9_	8.0	353.0880	353.0878	0.5	**191.0568**	5-*O-*caffeoylquinic acid (2)
**4**	5.658	C_17_H_20_O_9_	8.0	367.1043	367.1035	2.3	193.0506, **191.0561**, 173.0482, 134.0372, 93.0349	5-*O-*feruloylquinic acid
**5**	7.341	C_27_H_30_O_17_	13.0	625.1410	625.1410	0.0	**625.1410**, 301.0354, 300.0275, 271.0243, 255.0296, 178.9995, 151.0045	Quercetin-3-*O*-dihexoside
**6**	7.503	C_26_H_28_O_16_	13.0	595.1307	595.1305	0.4	**595.1307**, 301.0354, 300.0276, 271.0253, 255.0288	Quercetin-3*-O*-hexosylpentoside
**7**	7.751	C_27_H_30_O_16_	13.0	609.1463	609.1461	0.3	609.1461, 301.03547, **300.0277**, 271.0250, 255.0302	Quercetin-3-*O*-hexosyldeoxyhexoside (1)
**8**	7.848	C_21_H_20_O_12_	12.0	463.0885	463.0882	0.6	463.0885, 301.0354, **300.0376**, 271.0243, 255.0294	Quercetin-3*-O*-hexoside
**9**	8.197	C_27_H_30_O_16_	13.0	609.1463	609.1461	0.3	609.1463, 301.0354, **300.0276**, 271.0244, 255.0296, 243.0299, 178.9994, 151.0051	Quercetin-3-*O*-hexosyldeoxyhexoside (2)
**10**	8.674	C_28_H_32_O_17_	13.0	639.1562	639.1567	−0.7	639.1562, **315.0510**, 314.0431, 300.0264, 299.0197, 271.0253	Isorhamnetin-3*-O*-dihexoside
**11**	8.889	C_21_H_20_O_11_	12.0	447.0935	447.0933	0.5	447.0935, 327.0525, 285.0405, **284.0326**, 255.0304, 227.0353, 174.9581	Kaempferol-3-*O*-hexoside (1)
**12**	8.928	C_24_H_22_O_15_	14.0	549.0888	549.0886	0.4	505.0988, 463.0885, 301.0354, **300.0284**, 271.0255, 255.0306, 243.0293	Quercetin-3-*O*-malonylhexoside
**13**	9.147	C_25_H_24_O_12_	14.0	515.1196	515.1195	0.2	353.0878, **191.0561**, 179.0347, 135.0460	3,5-di-*O*-caffeoylquinic acid
**14**	9.446	C_27_H_30_O_15_	13.0	593.1515	593.1512	0.5	593.1515, **285.0405**, 284.0320, 255.0297, 227.0347	Kaempferol-7*-O-*rutinoside
**15**	9.484	C_21_H_20_O_11_	12.0	447.0935	447.0933	0.5	447.0935, 327.0487, 285.0405, **284.0325**, 255.0297, 227.0353, 151.0026	Kaempferol-3-*O*-hexoside (2)
**16**	9.544	C_28_H_32_O_16_	13.0	623.1618	623.1618	0.1	**623.1618**, 315.0510, 314.0433, 299.0207, 271.0254	Isorhamnetin hexosyldeoxyhexoside (1)
**17**	9.740	C_22_H_22_O_12_	12.0	477.1040	477.1039	0.3	477.1040, 315.0510, **314.0441**, 300.0265, 299.0200, 285.0420, 271.0258, 243.0313, 151.0065	Isorhamnetin-3-*O*-hexoside (1)
**18**	9.937	C_28_H_32_O_16_	13.0	623.1618	623.1618	0.1	623.1618, **315.0510**, 314.0430, 300.0272, 299.0195, 271.0245	Isorhamnetin-3-*O*-hexosyldeoxyhexoside (2)
**19**	10.015	C_22_H_22_O_12_	12.0	477.1040	477.1039	0.3	477.1040, 357.0603, 315.0510, **314.0428**, 300.0264, 299.0185, 285.0402, 271.0243, 257.0453, 243.0297	Isorhamnetin-3-*O*-hexoside (2)
**20**	11.147	C_25_H_24_O_15_	14.0	563.1042	563.1042	−0.1	519.1181, **315.0510**, 314.0428, 300.0269, 299.0194, 271.0244, 255.0278, 243.0286	Isorhamnetin-3-*O*-malonylhexoside
**21**	15.159	C_47_H_74_O_19_	11.0	941.4790	941.4752	4.1	941.4790, **779.4207**, 717.4229, 629.3699, 585.3797, 471.3480, 407.3319	3*-O-(*pentosyl)hexuronidyl 28-*O*-echinocystic acid hexosyl ester
**22**	15.440	C_48_H_78_O_19_	10.0	1003.5119 *	1003.5119	0.0	**795.4543**, 733.4525, 633.3981, 615.3903, 471.3419, 407.3305, 119.0342, 113.0236, 101.0237, 89.0232	3-*O*-dihexosyl 28-*O*-echinocystic acid hexosyl ester (1)
**23**	15.635	C_42_H_66_O_15_	10.0	809.4369	809.4329	4.9	809.4337, 689.3950, 647.3855, 585.3819, 513.3615, 471.3484, **407.3309**, 391.3031	3-*O*-hexuronidyl 28-*O*-echinocystic acid hexosyl ester
**24**	16.099	C_42_H_68_O_14_	8.0	841.4633*	841.4591	5.0	**633.4030**, 587.3957, 407.3348, 101.0241	3-*O*-hexosyl 28-*O*-echinocystic acid hexosyl ester
**25**	16.490	C_44_H_68_O_16_	11.0	851.4455	851.4435	2.4	809.4329, 689.3922, 585.3859, 539.3794, 471.3502, **407.3337**, 113.0238	3*-O*-hexuronidyl 28-*O-*echinocystic acid acetylhexosyl ester
**26**	16.635	C_48_H_74_O_19_	12.0	953.4792	953.4752	4.2	809.4329, 689.3908, **647.3816**, 629.3727, 585.3792, 471.3464, 407.3301, 113.0232	3-*O*-(hydroxymethylglutarylhexosyl)hexuronidyl echinocystic acid
**27**	17.009	C_54_H_88_O_23_	10.0	1149.5700 *	1149.5698	0.2	941.5115, 779.4576, 617.4046, 599.3933, 581.3822, 551,3725, **455.3506**	3-*O*-trihexosyl 28-*O*-oleanolic acid hexosyl ester
**28**	17.524	C_48_H_78_O_19_	10.0	1003.5100 *	1003.5119	−1.9	957.5079, 795.4570, 777.4456, 733.4562, 633.4042, 615.3939, 567.3686, **471.3495**, 407.3321, 113.0234, 101.0237	3-*O*-dihexosyl 28-*O*-echinocystic acid hexosyl ester (2)
**29**	17.683	C_47_H_74_O_18_	11.0	925.4810	925.4802	0.9	925.4802, 763.4253, 719.4352, **701.4265**, 629.4045, 613.3731, 587.3929, 569.3839, 551.3730, 523.3779, 497.3614, 455.3506, 453.3341, 437.3398, 423.3254, 407.3281	3*-O*-(pentosyl)hexuronidyl 28*-O*-oleanolic acid hexosyl ester (1)
**30**	18.020	C_48_H_78_O_18_	9.0	987.5218 *	987.5170	4.8	779.4587, **617.4060**, 599.39409, 455.3512	3-*O*-dihexosyl 28*-O*-oleanolic acid hexosyl ester
**31**	18.395	C_47_H_74_O_18_	11.0	925.4812	925.4802	1.0	925.4812, 763.4269, 701.4293, 629.4091, **613.3749**, 569.3854, 523.3827, 497.3650, 455.3526, 453.3475	3-*O*-(pentosyl)hexuronidyl 28-*O*-oleanolic acid hexosyl ester (2)
**32**	18.475	C_42_H_66_O_14_	10.0	793.4390	793.4380	1.3	793.4390, 631.3866, 569.3860, 497.3633, **455.3523**, 437.3403, 113.0240	3-*O*-hexuronidyl 28-*O*-oleanolic acid hexosyl ester
**33**	18.973	C_42_H_68_O_13_	8.0	825.4687 *	825.4652	4.7	**617.4069**	3-*O*-hexosyl 28*-O*-oleanolic acid hexosyl ester (1)
**34**	19.473	C_44_H_68_O_15_	11.0	835.4501	835.4485	1.9	793.4390, 673.3993, 613.3799, **569.3885**, 551.3763, 497.3660, 455.3536, 437.3458	3-*O-*hexuronidyl 28-*O*-oleanolic acid acetylhexosyl ester
**35**	19.633	C_48_H_74_O_18_	12.0	937.4812	937.4802	1.0	793.4390, 673.39682, 631.3862, 613.3753, **569.3851**, 497.3628, 455.3520, 437.3411	3-*O*-(hydroxymethylglutaryl)hexuronidyl 28-*O*-oleanolic acid hexosyl ester
**36**	20.423	C_42_H_64_O_14_	11.0	791.4235	791.4223	1.5	647.3791, 471.3480, **407.3286**, 391.2967, 113.0225	3-*O*-(hydroxymethylglutaryl)hexuronidyl echinocystic acid
**37**	22.256	C_41_H_64_O_13_	10.0	763.4303	763.4274	3.8	763.4303, 613.3767, 569.3879, 537.3592, 497.3602, 455.3531, **453.3356**, 437.3426, 407.3333	3-*O*-(pentosyl)hexuronidyl oleanolic acid
**38**	22.615	C_42_H_68_O_13_	8.0	825.4662 *	825.4652	2.8	**617.4059**, 599.3959, 455.3528, 113.0231, 101.0235	3-*O*-hexosyl 28-*O-*oleanolic acid hexosyl ester (2)

## Data Availability

Not applicable.

## Supplementary Materials

The following supporting information can be downloaded at: https://www.mdpi.com/article/10.3390/molecules28010199/s1. Figure S1. TOF-MS/MS spectra of compounds (**a**) **12** and (**b**) **20**, and (**c**) their fragmentation pathways; Figure S2. (**a**) TOF-MS/MS spectrum of compound **21**; (**b**) its hypothesized fragmentation pathway; Figure S3. (**a**) TOF-MS/MS spectrum of compound **25**; (**b**) its hypothesized fragmentation pathway; Figure S4. (**a**) TOF-MS/MS spectrum of compound **24**; (**b**) its hypothesized fragmentation pathway; Figure S5. TOF-MS/MS spectra of compounds (**a**) **22** and (**b**) **28**, and (**c**) the putative fragmentation pathway; Figure S6. TOF-MS/MS spectra of compounds (**a**) **29** and (**b**) **31**, and (**c**) the putative fragmentation pathway; Figure S7. (**a**) TOF-MS/MS spectrum of compound **37**; (**b**) its hypothesized fragmentation pathway; Figure S8. Proposed biotransformation pattern for glycosylated flavonols during in vitro simulated digestion.

## Sample Availability

Samples of the investigated extracts are available from the authors.
