# Supplementary material for: Encapsulating Calendula arvensis (Vaill.) L. Florets: UHPLC-HRMS Insights into Bioactive Compounds Preservation and Oral Bioaccessibility
Source: Molecules. 2022 Dec 26;28(1):199. doi: 10.3390/molecules28010199 (PMC9822028; doi:10.3390/molecules28010199)
Supplement: Supplementary file 1 [file molecules-28-00199-s001.zip › molecules-2122401-supplementary.pdf]

Supplementary materials

Encapsulating *Calendula arvensis* (Vaill.) L. Florets: UHPLC-HRMS Insights into Bioactive Compounds Preservation and Oral Bioaccessibility

Marika Fiorentino [1], Simona Piccolella [1], Claudia Gravina [1], Adriano Stinca [1], Assunta Esposito [1,*], Michelina Catauro [2], Severina Pacifico [1,*]

[1] Department of Environmental, Biological and Pharmaceutical Sciences and Technologies, University of Campania "Luigi Vanvitelli" Via Vivaldi, 43 81100 Caserta; marika.fiorentino@unicampania.it (M.F.); simona.piccolella@unicampania.it (S.P.); claudia.gravina@unicampania.it (C.G.); adriano.stinca@unicampania.it (A.S.); assunta.esposito@unicampania.it (A.E.); severina.pacifico@unicampania.it (S.P.)

[2] Department of Engineering, University of Campania "Luigi Vanvitelli", Via Roma 29, I-813031, Aversa, Italy michelina.cataurо@unicampania.it

Correspondence: severina.pacifico@unicampania.it; assunta.esposito@unicampania.it

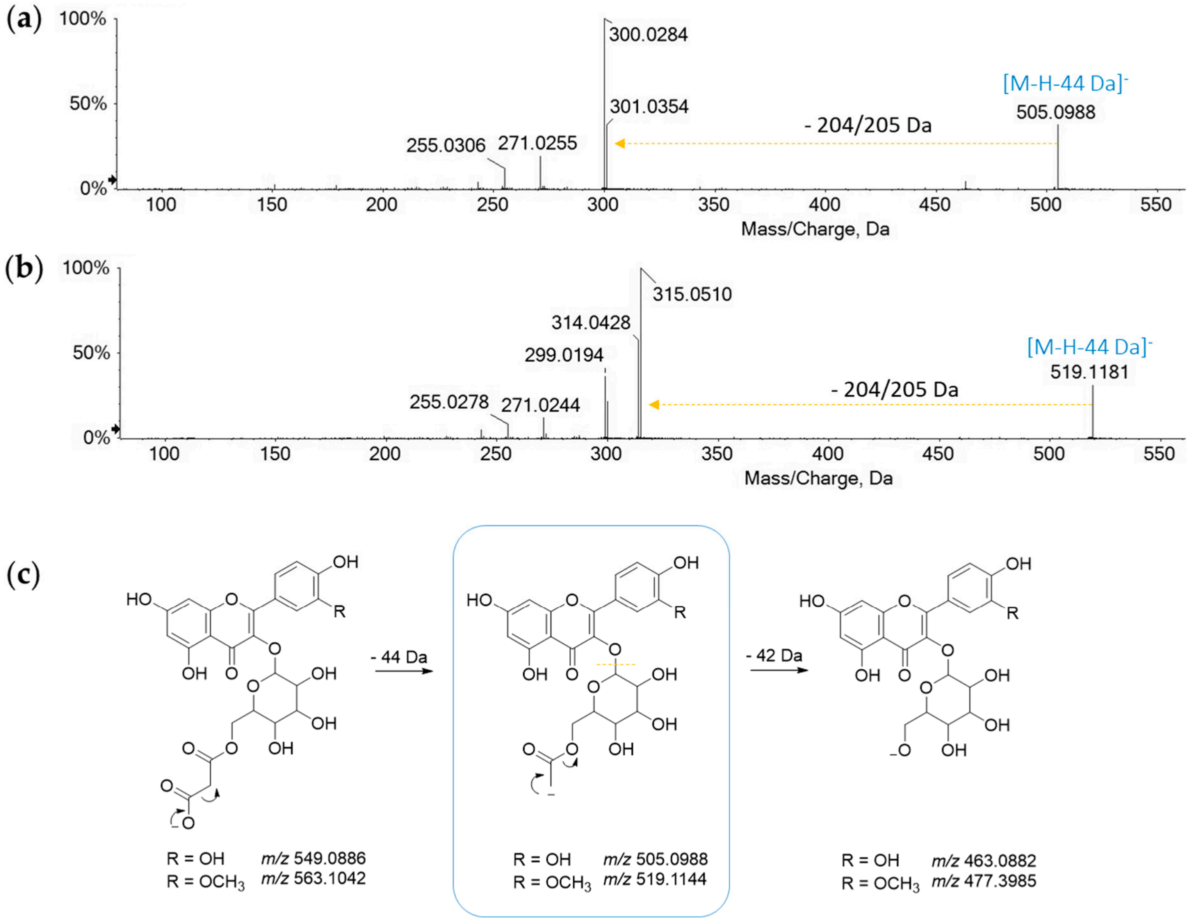

Figure S1. TOF-MS/MS spectra of compounds (**a**) **12** and (**b**) **20,** and (**c**) their fragmentation pathways.

(**a**)

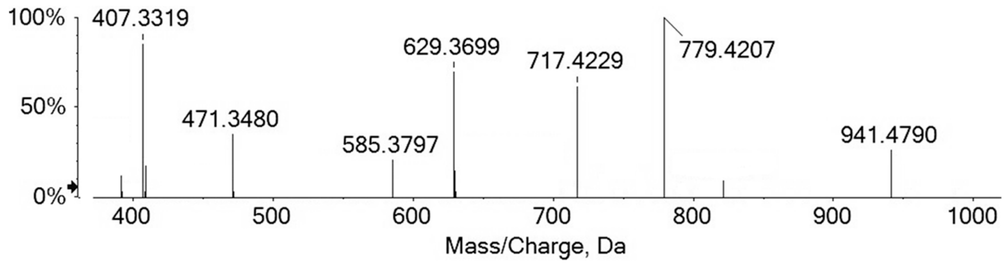

(**b**)

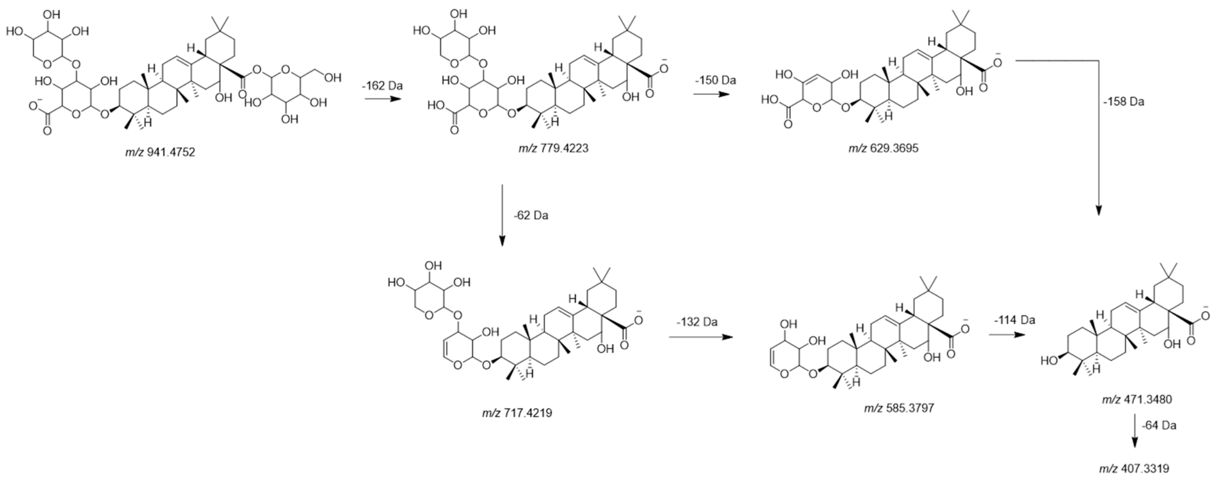

Figure S2. (**a**) TOF-MS/MS spectrum of compound **21**; (**b**) its hypothesized fragmentation pathway.

(a)

(b)

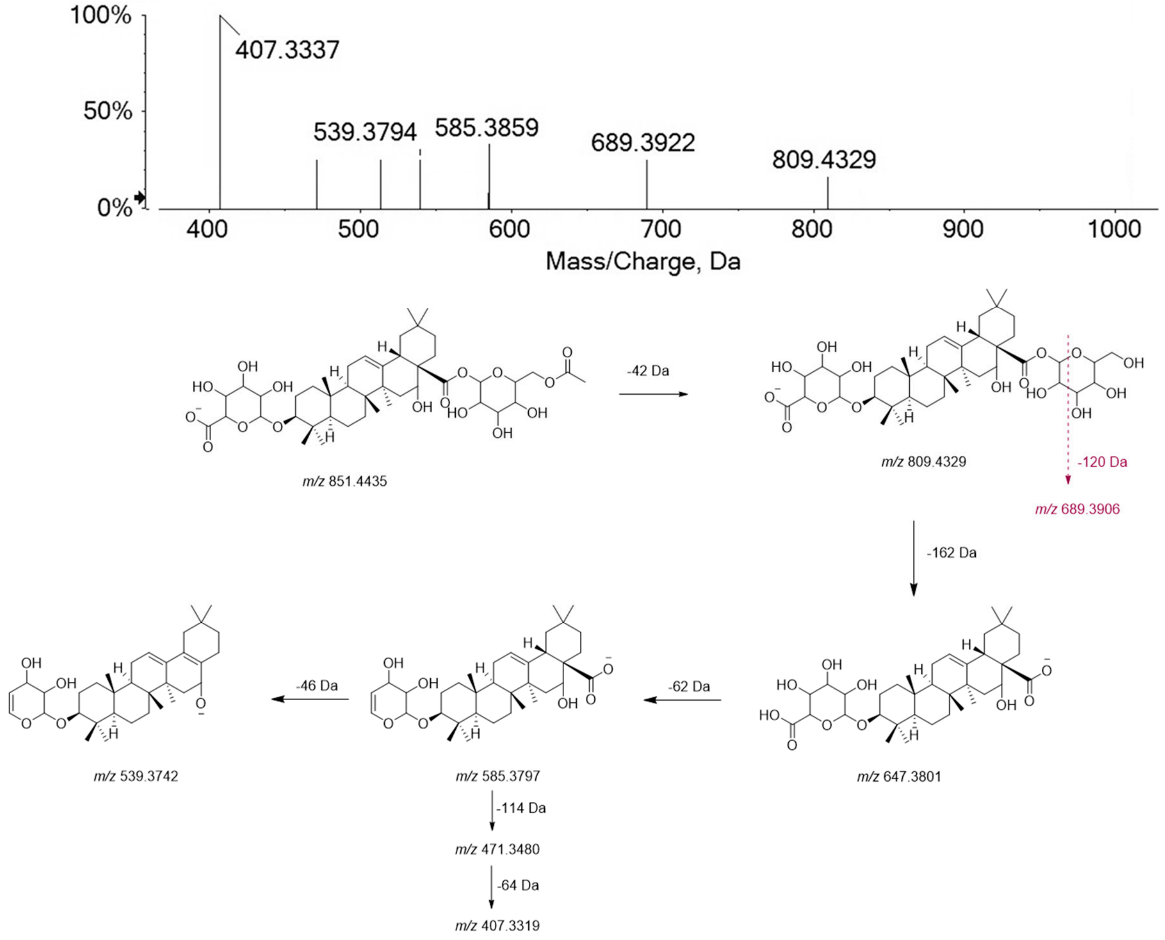

Figure S3. (**a**) TOF-MS/MS spectrum of compound **25**; (**b**) its hypothesized fragmentation pathway.

(a)

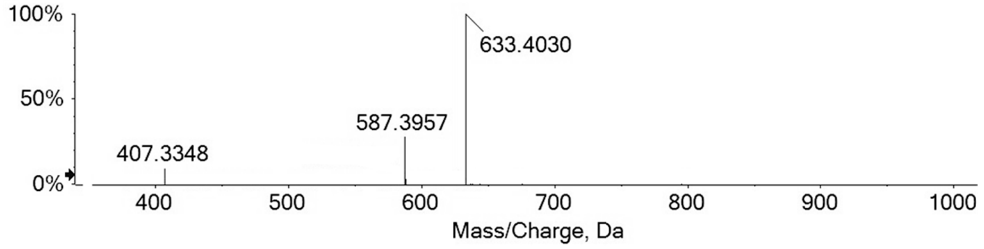

(b)

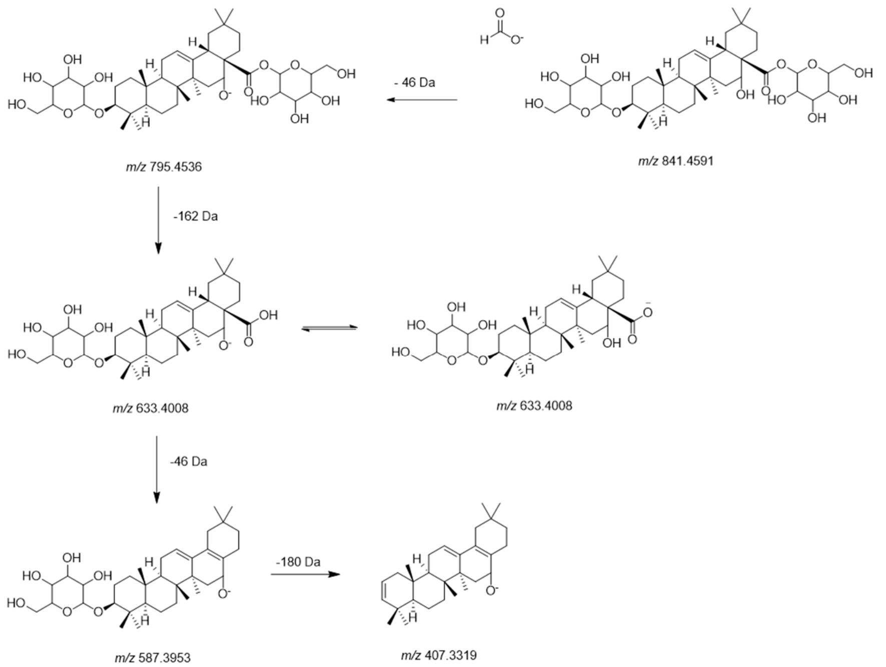

Figure S4. (**a**) TOF-MS/MS spectrum of compound **24**; (**b**) its hypothesized fragmentation pathway.

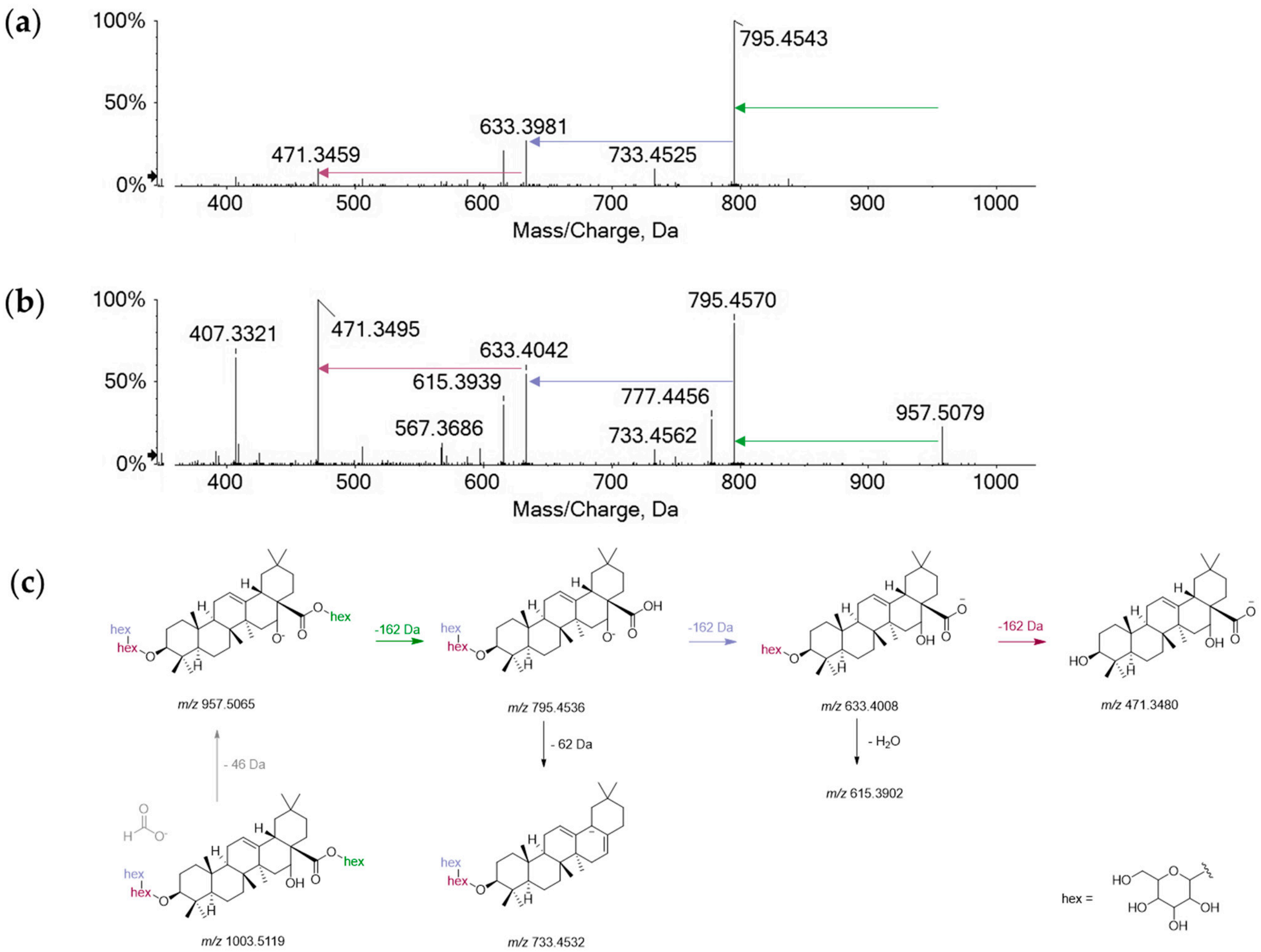

Figure S5. TOF-MS/MS spectra of compounds (**a**) **22** and (**b**) **28,** and (**c**) the putative fragmentation pathway.

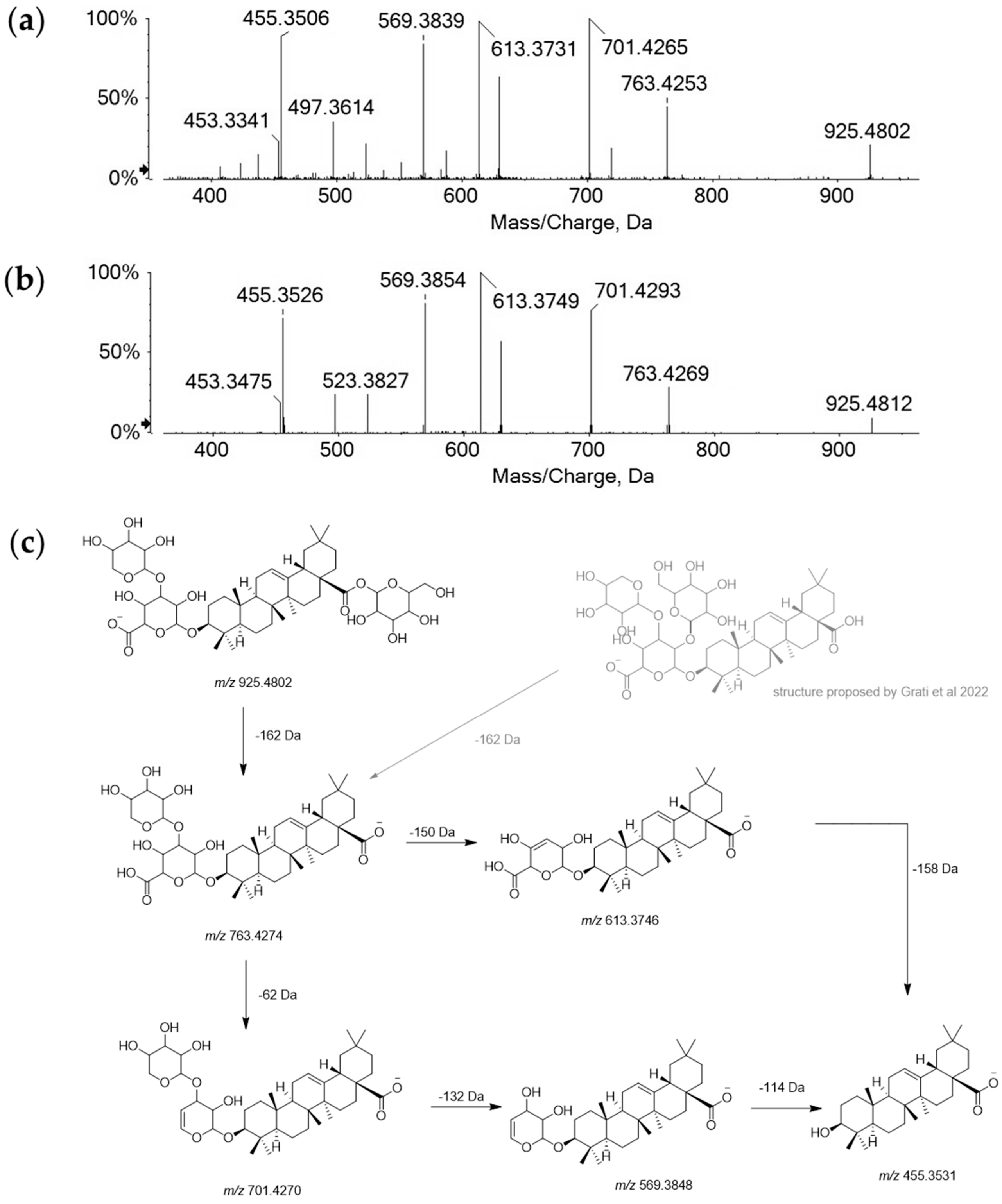

Figure S6. TOF-MS/MS spectra of compounds (**a**) **29** and (**b**) **31,** and (**c**) the putative fragmentation pathway.

(**a**)

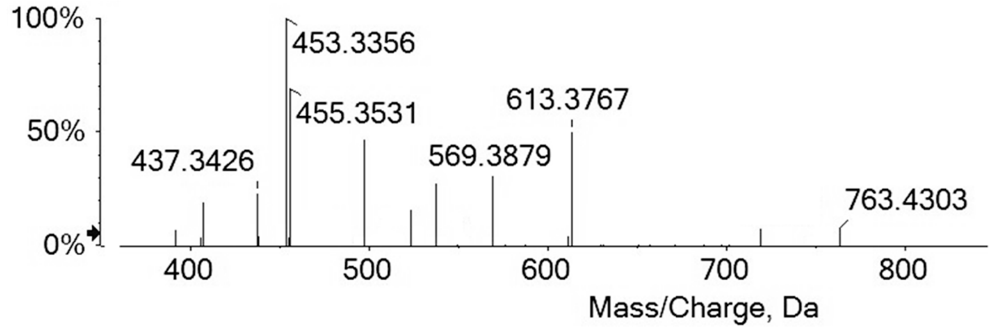

(**b**)

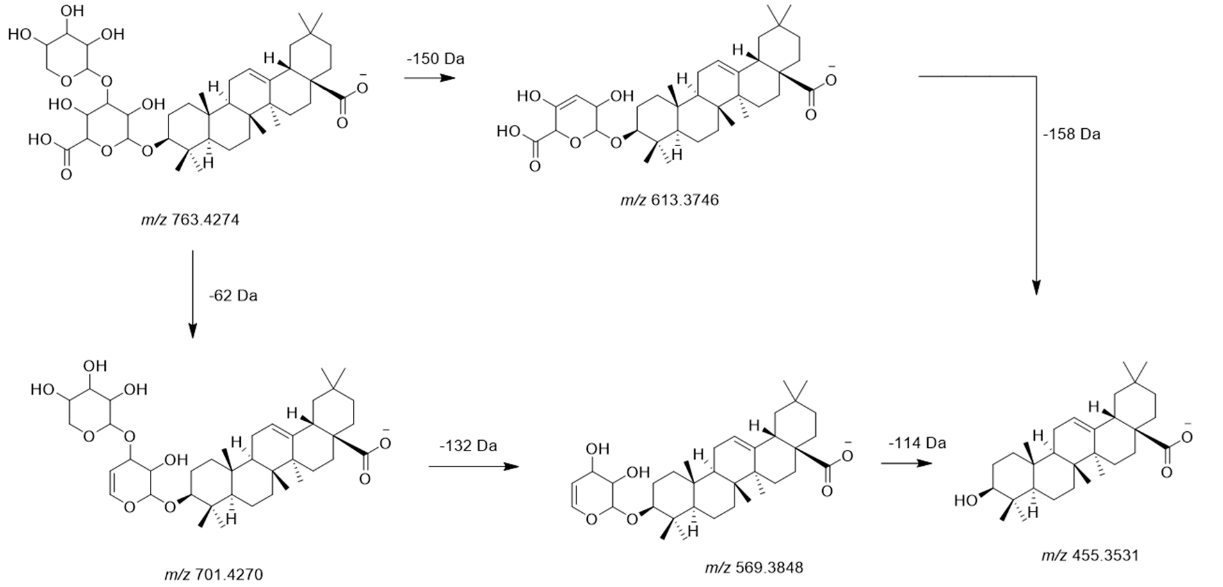

Figure S7. (**a**) TOF-MS/MS spectrum of compound **37**; (**b**) its hypothesized fragmentation pathway.

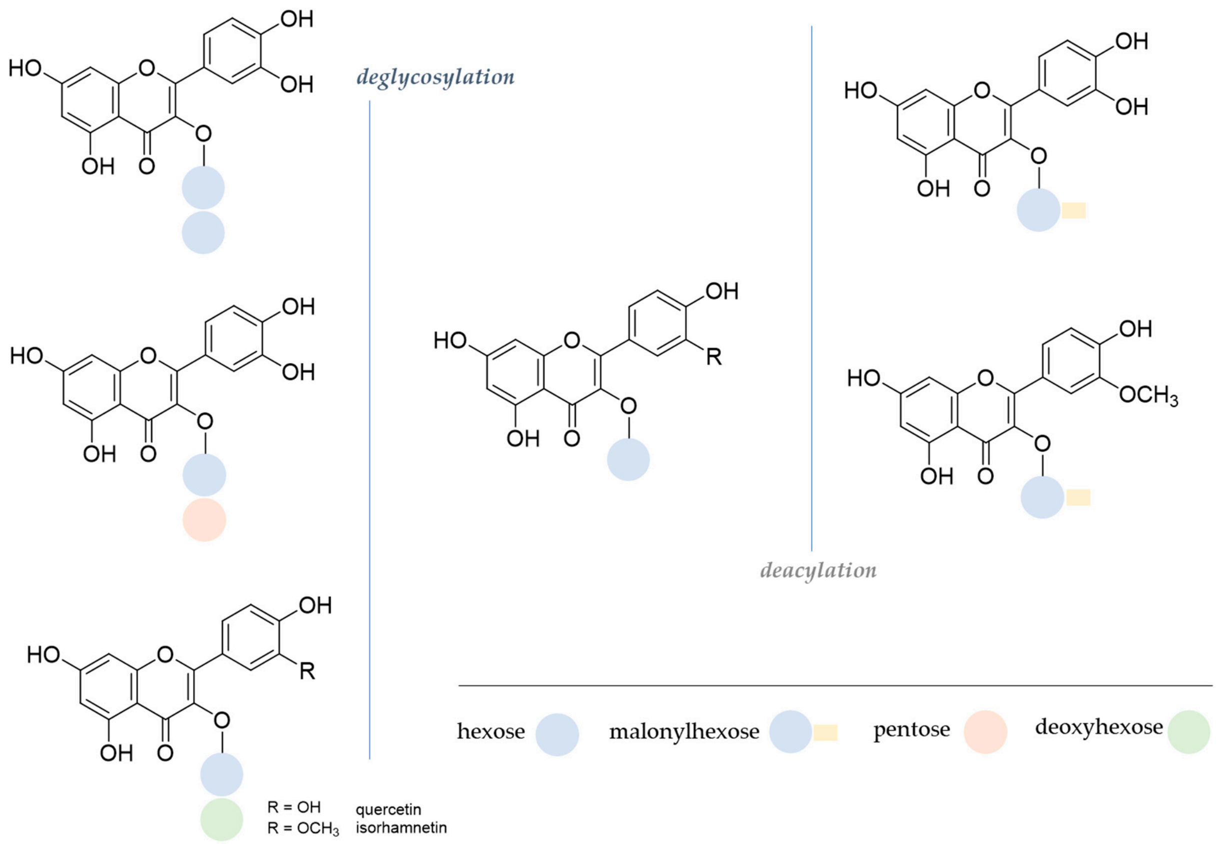

Figure S8. Proposed biotransformation pattern for glycosylated flavonols *during* in vitro *simulated* digestion.
